# Nicotine Exposure During Pregnancy and Postnatal Cognitive Outcomes: A Systematic Review and Meta‐Analysis

**DOI:** 10.1111/apa.70539

**Published:** 2026-04-20

**Authors:** Deimantė Baguckaitė, Judith Covey, Nadja Reissland

**Affiliations:** ^1^ Department of Psychology Durham University Durham UK

**Keywords:** cognition, meta‐analysis, nicotine exposure, pregnancy, review

## Abstract

**Aim:**

Nicotine exposure during pregnancy interferes with critical periods of foetal brain development, disrupting the timing and functioning of neurodevelopment. This systematic review and meta‐analysis aims to evaluate the effect of prenatal nicotine exposure on postnatal cognitive outcomes.

**Methods:**

Web of Science, PubMed, PsycINFOand OpenGrey databases were searched, with no date restrictions. Human (k = 1) and animal research (k = 8) was included. Studies were required to include at least one postnatal cognitive measure during the pre‐weaning period.

**Results:**

Exposure to nicotine during pregnancy negatively impacted postnatal outcomes of spatial memory (Cohen's *d* = −1.117; 95% CI [−2.112, −0.123]), centre exploration in animal studies (*d* = −1.054; 95% CI [−1.659, −0.449]), and reflex development in human and animal studies (*d* = −1.126; 95% CI [−1.737, −0.514]). Prenatal nicotine exposure did not have a significant effect on ambulation (*d* = −0.044; 95% CI [0.485, 0.397]) and rearing (*d* = −0.45; 95% CI [−1.042, 0.142]) in animals.

**Conclusion:**

Findings suggest that certain domains may be especially vulnerable to prenatal nicotine exposure, with reflex development showing the strongest effect. The scarcity of human studies underscores an urgent need for further research to ensure informed recommendations on nicotine intake during pregnancy.

## Summary

- Nicotine delivery system use during pregnancy exposes the foetus to nicotine during critical periods of brain development.
- Animal studies, supported by one human study, show that prenatal nicotine exposure is linked with negative postnatal outcomes, with the largest effect observed in the development of reflexes.
- While human evidence on cognitive impacts remains limited, further research is essential to fully inform on the safety of nicotine intake during pregnancy, particularly amid growing e‐cigarette use.

**Abbreviations**
CI: Confidence Interval
CNS: Central Nervous System
nAChRs: Nicotinic Acetylcholine Receptors
NBAS: Neonatal Behavioural Assessment Scale
NRT: Nicotine Replacement Therapy

## 1 Introduction

Nicotine replacement therapy (NRT) is widely used in an attempt to manage the addictive cravings associated with smoking, and to facilitate cessation [1]. More recently, e‐cigarettes have emerged as an alternative nicotine delivery method, often marketed with claims of supporting smoking cessation [2]. In England, current pregnancy health care guidance supports the use of NRT products and recommends e‐cigarettes as a safer alternative to traditional cigarettes, due to the absence of carbon monoxide and tar [3]. However, this approach is not globally accepted; other international health organisations, such as the Centers for Disease Control and Prevention, instead emphasise complete abstinence from all forms of nicotine during pregnancy [4].

Nicotine readily passes into the foetal environment via the placenta and freely crosses the blood–brain barrier [5], shown to interfere with the physiological and cognitive development of the foetus [6, 7]. Evidence also suggests that prenatal exposure to nicotine via non‐combustible products, such as snuff, can increase the risk of sudden infant death syndrome [8]. Nicotinic acetylcholine receptors (nAChRs) are present in the foetal brain from early gestation at the neural tube stage and are crucial for processes of brain development [9]. In the Central Nervous System (CNS), nAChRs are involved in critical developmental processes, including neurogenesis, apoptosis and cell proliferation [10]. Animal research has demonstrated that prenatal nicotine exposure can disrupt foetal brain development, by interfering with the programming and timing of neurodevelopmental events [11]. Given prenatal nicotine exposure interferes with processes during critical developmental periods, this can have long‐lasting detrimental effects on cognition beyond the prenatal period [12, 13].

An emerging concern is the increasing prevalence of e‐cigarette use in the UK, including among individuals who have never regularly smoked [14]. Surveys in England and Scotland report that 5.2% of pregnant women use e‐cigarettes exclusively in late pregnancy [15]. This mirrors trends in the USA where exclusive e‐cigarette use during pregnancy has increased from 0.4% to 0.6% since 2016 to 2019 [16]. The higher prevalence of vaping in the UK may reflect the public perception that e‐cigarettes are a safer alternative to cigarette smoking, reinforced by healthcare guidance. Given the rising rates of exclusive e‐cigarette use and widespread availability of NRT, it is essential to evaluate the effects of nicotine exposure per se, rather than comparing products solely to cigarette smoking.

The current systematic review and meta‐analysis investigate the effects of prenatal nicotine exposure on postnatal cognitive development. Existing reviews of nicotine exposure during pregnancy have primarily focused on physical growth outcomes, including birthweight or lung development [17, 18]. Cognitive outcomes are examined less consistently and most often within reviews assessing broader pregnancy outcomes of physical and clinical impacts of e‐cigarette use on foetal, infant and maternal health [19, 20], as well as social perceptions surrounding vaping [19]. Additionally, animal research is often excluded, despite offering valuable insights from experimental studies that are ethically not feasible in research conducted on humans. One existing review included human and animal research to narratively examine neurodevelopment and brain growth; however, postnatal cognitive outcomes were not the primary emphasis [21].

To address these gaps in the literature, the present review synthesises findings from both animal and human studies to assess the impact of prenatal nicotine exposure on postnatal outcomes in offspring. By focusing on specific cognitive domains, we were able to conduct a meta‐analysis, providing a rigorous, quantitative summary of current evidence.

## 2 Methodology

The systematic review and meta‐analysis reported in this paper follows PRISMA guidelines [22].

### 2.1 Search Strategy

A literature search of four databases was conducted (Web of Science Core Collections, PubMed, PsycINFO and OpenGrey), with no beginning date restrictions to August 2024. The search terms are listed in (Table 1).

**TABLE 1:** Search terms.

Prenatal terms	AND	Nicotine exposure terms	AND	Postnatal terms	AND	Cognitive terms	NOT	Exclusion terms
Prenatal*		E‐cig*		Postnatal*		Cognition		Tobacco
OR		OR		OR		OR		OR
Pregnancy		Electronic cigarette*		Post‐partum		Cognitive*		Cannabis
OR		OR		OR		OR		OR
Pregnant wom*		Electronic nicotine delivery system		Post‐pregnancy		Behaviour*		Alcohol
OR		OR		OR		OR		OR
Mother*		Vaping*		Neonate*		Sensory		Cocaine
OR		OR		OR		OR		OR
Maternal		Nicotine*		Newborn*		Development*		Adult*
OR				OR		OR		OR
Fetal				Baby		Processing		Adolescent*
OR				OR		OR		OR
Fetus*				Babies		Memory		Asthma*
OR				OR		OR		
Foetal				Infant*		Learning		
OR				OR		OR		
Foetus*				Child*		Recognition		
OR				OR		OR		
Womb				Offspring*		Attention		
OR						OR		
Intrauterine						Perception		
OR						OR		
Perinatal*						Reflex*		

*Note:* No date restriction was applied in the article search strategy. “AND” indicates studies were required to have one term from each column, “OR” indicates any of the terms within a column are adequate for eligibility, “NOT” indicates exclusion if any listed term in this column was present. * Indicates truncation in search terms.

### 2.2 Screening of Studies

All database search results were combined. Duplicate records, as well as any reviews (literature, systematic and meta‐analyses) were removed. The first round of study screening was completed by the first author (DB) and consisted of title and abstract review. The full‐text articles were screened by the first author for eligibility according to the inclusion criteria. Additionally, the reference lists of reviews, and research articles which remained from the first screening, were manually searched to identify potentially relevant research. Research conducted with both placental mammalian animals and humans was included. Animal studies met the inclusion criteria of the systematic review if the study used a randomised experimental design to compare a group of offspring exposed prenatally to nicotine and a non‐exposed control or comparison group. In human studies, only quasi‐experimental designs were eligible for inclusion, which for ethical reasons relied on comparing naturally occurring groups of offspring whose mothers reported nicotine intake during pregnancy through any delivery system apart from traditional cigarettes (e.g., NRT and e‐cigarettes) with a control group not exposed to any form of nicotine.

Studies where nicotine exposure occurred at any stage of gestation in both humans and animals were eligible. There were no criteria that specified continuous exposure throughout the full duration of pregnancy. Because nicotine can be transferred to the newborn through breastfeeding [23], we did not exclude studies where the mother was exposed to nicotine postnatally as well as prenatally. However, animal studies which directly exposed the offspring to nicotine postnatally were excluded from the review. At least one postnatal cognitive measure was required in the included studies, characterised as observable, performance‐based behaviours, such as memory performance (e.g., recognition of novelty in animal studies), early demonstration of reflexes (e.g., righting reflex in animal studies or Moro reflex in humans) and exploratory activity (e.g., rearing in animal studies). Studies were included if they carried out measures in pre‐weanling animals, or at 6 months of age or younger in human studies, corresponding to the approximate period when the offspring would be reliant on maternal or formula feeding. Inclusion criteria were restricted to the pre‐weaning period, as it provides a defined and consistent window which allows a comparison of animal and human studies, thereby reducing heterogeneity in study outcomes. There are no existing reviews which have systematically investigated early cognitive outcomes of nicotine exposure, and by focusing on pre‐weanling animals and offspring under 6 months of age, we can identify early developmental impacts which may reflect indicators of long‐term cognitive and behavioural outcomes beyond the prenatal period [13, 24].

The full text of articles which remained after the first round of screening were also reviewed in accordance with the eligibility criteria by a reliability coder, who was a student affiliated with the Fetal and Neonatal Research Laboratory at Durham University. Disagreements were resolved by a reliability reviewer, who was the third author (NR).

### 2.3 Data Extraction

Data were extracted using a pre‐defined record sheet, with information including the nature of the control or comparison conditions, sample characteristics (species, sample size, offspring age and if prenatally nicotine‐exposed offspring were cross‐reared to a nicotine naïve dam), nicotine exposure during pregnancy (dosage, duration and route of nicotine exposure including: infusion, subcutaneous, intravenous or oral administration), and outcome measures with effect sizes and standard errors of the effect sizes. Where an effect size (Cohen's d) was not provided in the articles, it was calculated using the data available with use of the Campbell Collaboration effect size calculator (https://www.campbellcollaboration.org/calculator/). If measures were obtained at multiple time points, effect sizes were recorded for the first available time point, with no minimum age restriction for offspring at the time of testing. This approach allowed us to investigate whether variation in effect sizes was influenced by age at which offspring were first tested, acknowledging that a common testing age could not be identified across all studies.

### 2.4 Assessment of Risk of Bias

The ROBINS‐I tool [25], was used to determine the risk of bias for the studies included in this review that had been conducted on humans using non‐randomised or quasi‐experimental designs. The SYRCLE Risk of Bias tool [26], was used to assess risk of bias in the animal studies. However, due to insufficient methodological reporting, all assessments were rated as “unclear”, and the tool did not help us to differentiate between the methodological quality of the studies. For example, no information was provided regarding research blinding or methods of randomisation of animals to different conditions. Risk of bias was assessed by the first author (DB) and reviewed by the third author (NR).

### 2.5 Data Analysis

The effect sizes from eligible studies were categorised according to different cognitive measures. Three main outcomes were identified (exploratory behaviour, spatial memory, and reflex development). Exploratory behaviour refers to information‐gathering or stimulus‐seeking behaviour, such as rearing in rodents. Spatial memory is defined by the process of encoding and recalling spatial information about the environment. Reflex development, including the Moro reflex in humans or righting reflex in animals, is observed as an indicator of early neurodevelopment.

The size of the effect was evaluated using Cohen's convention, with 0.2 indicating a small effect, 0.5 indicating a moderate effect and 0.8 and over indicating a large effect [27]. A negative effect size indicates that the prenatally nicotine‐exposed group scored lower on the outcome in comparison to the non‐exposed control group. If there was insufficient data provided in the article to calculate an effect size, authors were contacted. However, where additional information was not available, the measure was excluded from the meta‐analysis. If a study reported effect sizes separated by the sex of the sample, this was combined into a single overall effect size. This approach was taken because rats and mice are not sexually mature as pre‐weanlings [28], and analysis of sex differences would not be meaningful. Heterogeneity of effect sizes was assessed to indicate whether a fixed‐effects or random‐effects model should be reported. Heterogeneity was identified if the *p*‐value for Cochran's Q was significant (*p* < 0.05) [29]. Sensitivity analyses were undertaken to evaluate the impact of particular studies on the overall effect size [30]. The number of studies which would be required for retrieval for the effect size to be non‐significant was estimated using Rosenthal's Fail‐safe N [31]. Funnel plots could not be generated to explore small‐study effects, since our review included only nine studies instead of the required minimum of ten studies [32]. We had planned to use moderation analysis to identify potential moderators of effect size variability (e.g., age at which offspring were tested). However, this was not possible due to the insufficient number of studies which could be included in the review [30].

## 3 Results

### 3.1 Study Selection

**FIGURE 1:**
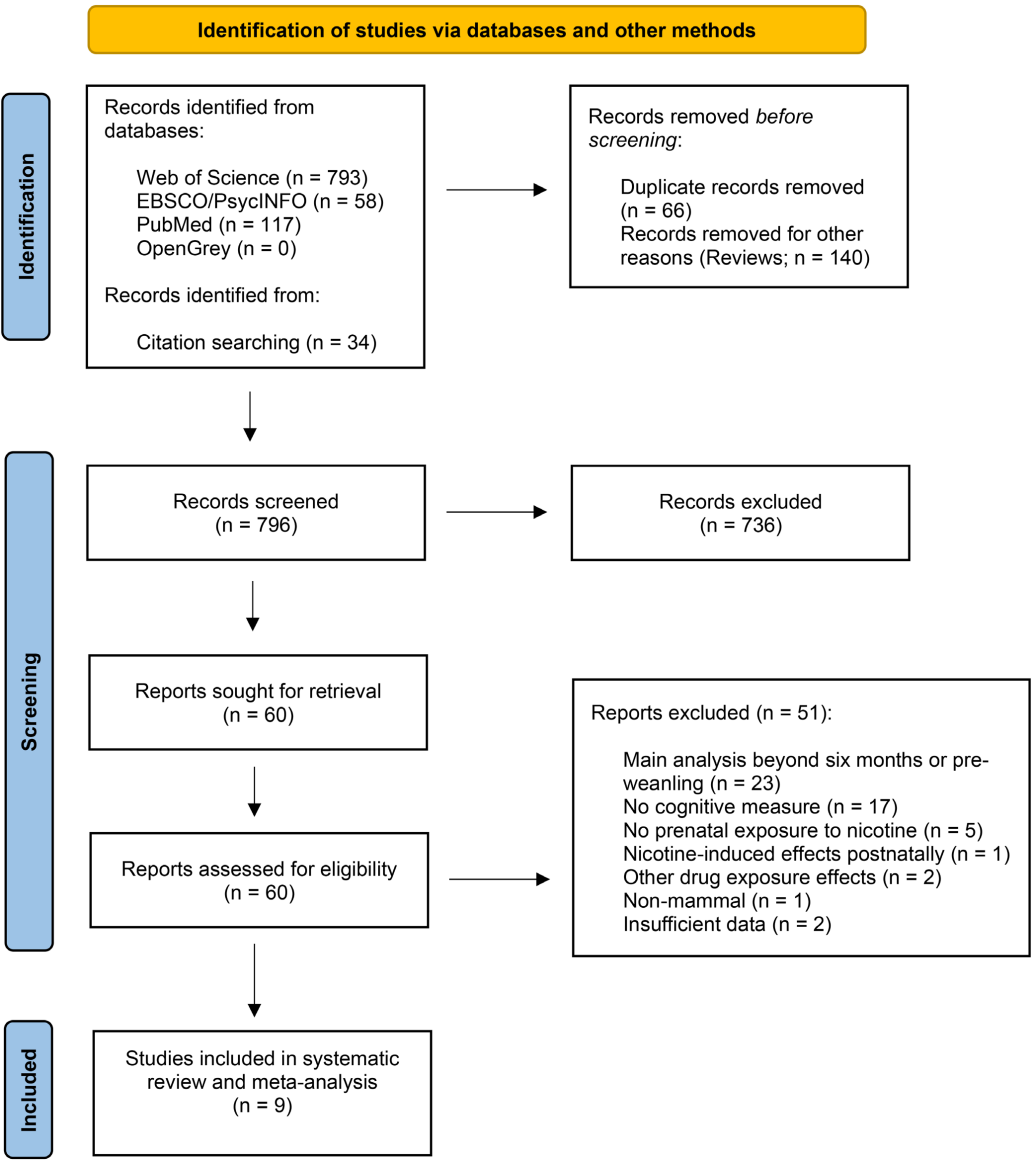
PRISMA 2020 flow diagram for new systematic reviews which included searches of databases, registers and other sources.

A total of 1002 potentially relevant articles were identified across the databases. After the removal of duplicates and ineligible records, 796 articles remained. Based on title and abstract screening, a total of 60 studies remained for full‐text evaluation. During this second round of screening, 51 articles were excluded, with nine studies meeting the eligibility criteria [33, 34, 35, 36, 37, 38, 39, 40, 41]. The most common reason for exclusion of articles was age of the sample when the postnatal measure was carried out. Other exclusion criteria included studies that lacked a postnatal cognitive measure, did not involve prenatal nicotine exposure, included direct postnatal exposure to nicotine, included co‐exposure with other substances, or had non‐mammalian samples. Two studies were excluded due to insufficient or unavailable data [42, 43]. See Figure 1 for the PRISMA flow diagram of study screening and selection.

### 3.2 Study Characteristics

Characteristics of the studies included in the review and meta‐analysis can be found in (Table 2).

**TABLE 2:** Studies included in the systematic review and meta‐analysis.

Reference (first author, year, country)	Sample	Nicotine exposure condition (route and dosage)	Control condition	Measure: effect size Cohen's d (SEd)
Ajarem 1997 [33] Saudi Arabia	14 mice pup scores (7 exposed, 7 not exposed) (1 score is equal to 3 pups) Mother‐reared, No cross‐rear	Exposure from GD9 to birth Daily subcutaneous injections (constant injectable volume of 0.1 mL per mouse) 0.5 mg/kg of body weight pure nicotine	Saline injection	REFLEX DEVELOPMENT (at PND1): Righting reflex: −1.663 (0.620) Rotating reflex: −1.343 (0.592)
Froggatt 2020 [34] UK	54 human infants (10 exposed, 44 not exposed)	E‐cigarette use Dosage unknown Unknown breast‐feeding exposure post‐birth	Non‐exposed infants	REFLEX DEVELOPMENT (at 1 month): NBAS reflex tests: −1.857 (0.393)
LeSage 2006 [35] USA	92 rats (52 exposed, 40 not exposed) Mother‐reared, No cross‐rear	Exposure from GD4 to birth Delivery of 0.03 mg/kg dose over 1 s via IV infusion pump in a volume of 50 μL for 16h a day at 14 min intervals, for a total dose of 2 mg/kg/day	IV saline infusion	EXPLORATORY BEHAVIOUR (at PND19): Ambulation: −0.349 (0.212) Rearing: −0.170 (0.211) Centre: Insufficient data to compute effect size
Muneoka 1997 [36] Japan	26 rats in infusion group, 26 rats in injection group (13 exposed, 13 not exposed in each group) Mother‐reared, No cross‐rear	Exposure from GD4 to GD20 Infusion group: 6 mg/kg/day osmotic minipump Injection group: 3 mg/kg twice daily	Sodium bitartrate Infusion (infusion group control) Saline injection (injection group control)	EXPLORATORY BEHAVIOUR (at PND14): Ambulation: Infusion group: 0.517 (0.159) Injection group: −0.060 (0.392) Rearing: Infusion group: −0.184 (0.393) Injection group: −0.099 (0.393)
Parameshwaran 2011 [37] USA	24 rats for exploratory behaviour measure (12 exposed, 12 not exposed), 18 rats for spatial memory measure (9 exposed, 9 not exposed) Cross‐rearing unclear	Exposure from GD3 to birth Infusion 6 mg/kg/day osmotic minipump	Saline infusion	EXPLORATORY BEHAVIOUR (at PND21): Ambulation: −0.849 (0.426) Centre: −1.223 (0.445) Rearing: −1.638 (0.472) SPATIAL MEMORY (at PND21): Novel arm visits: −0.975 (0.499) Novel arm duration: −1.260 (0.516)
Pauly 2004 [42] USA	20 mice (10 exposed, 10 not exposed) Yes cross‐reared	Exposure from GD0 and pre‐mating to birth 200 μg/mL in drinking water	2% saccharin water drinking solution	EXPLORATORY BEHAVIOUR (at PND20): Ambulation: Insufficient data to compute effect size Rearing: Insufficient data to compute effect size Not included in meta‐analysis
Schlumpf 1988 [38] Switzerland	24 nicotine‐exposed rats in no cross‐rear group, 16 nicotine‐exposed rats in cross‐reared group, 21 not exposed rats in control group	Exposure from GD12 to GD19 Infusion delivery at a rate of 25 μg/100 g per hour osmotic minipump	Tartaric acid dissolved in water infusion	EXPLORATORY BEHAVIOUR (at PND7): Ambulation: No cross‐rear group: −0.817 (0.354) Cross‐reared group: −1.899 (0.421)
Schneider 2010 [39] UK	40 nicotine‐exposed rats in 0.04 mg/mL group, 33 nicotine‐exposed rats in 0.06 mg/mL group, 12 nicotine‐exposed rats in 0.08 mg/mL group, 95 non‐exposed rats in control group Yes cross‐reared	Exposure from GD0 and pre‐mating to birth Nicotine in drinking water 0.04 mg/mL group 0.06 mg/mL group 0.08 mg/mL group	Drinking water	EXPLORATORY BEHAVIOUR (at PND19): Ambulation: 0.04 mg/mL group: 0.155 (0.189) 0.06 mg/mL group: 0.266 (0.205) 0.08 mg/mL group: 0.752 (0.313) REFLEX DEVELOPMENT (at PND2): Negative geotaxis: 0.04 mg/mL group: −0.920 (0.198) 0.06 mg/mL group: −0.629 (0.208) 0.08 mg/mL group: −2.094 (0.340) Righting reflex: 0.04 mg/mL group: 0.016 (0.189) 0.06 mg/mL group: −0.678 (0.209) 0.08 mg/mL group: −2.648 (0.368)
Shacka 1997 [43] USA	Unclear sample size of rats Yes cross‐reared	Exposure from GD7 to birth Infusion 2 mg/kg/day osmotic minipump	Saline infusion	EXPLORATORY BEHAVIOUR (at PND14): Ambulation: Insufficient data to compute an effect size REFLEX DEVELOPMENT (at PND2): Righting reflex: Insufficient data to compute an effect size Negative geotaxis: Insufficient data to compute effect size Not included in meta‐analysis
Sobrian 1995 [40] USA	26 rats (13 exposed, 13 non‐exposed) Mother‐reared, No cross‐rear	Exposure from GD8 to GD21 Injection 5 mg/kg/day	Saline injection	REFLEX DEVELOPMENT (at PND3): Righting reflex: −0.507 (0.399)
Zhou 2021 [41] China	24 mice for exploratory behaviour measure (12 exposed, 12 not exposed), 22 mice for auditory startle test (8 exposed, 14 not exposed), 31 mice for olfactory reflex test (17 exposed, 14 not exposed) Mother‐reared, No cross‐rear	Exposure from GD0 and pre‐mating and post‐birth exposure 200 μg/mL in drinking water	1% saccharin water drinking solution	EXPLORATORY BEHAVIOUR (at PND20): Ambulation: 1.640 (0.472) Centre: −0.897 (0.428) REFLEX DEVELOPMENT (at PND11): Auditory startle: −1.571 (0.503) Olfactory reflex: −1.654 (0.418)

Abbreviations: GD, Gestational day; PND, Postnatal day.

The studies included in the meta‐analysis were conducted in six different countries: UK (k = 2), Japan (k = 1), Switzerland (k = 1), China (k = 1), Saudi Arabia (k = 1), and USA (k = 3). The meta‐analysis included both human and animal research studies: human (k = 1), mice (k = 2), and rats (k = 6). In the human study [34], a total of 54 newborns aged one month were assessed, with 10 newborns prenatally exposed to nicotine via maternal e‐cigarette use during pregnancy. In the animal studies [33, 35, 36, 37, 38, 39, 40, 41], 544 offspring, of which 263 were not prenatally exposed to nicotine, were assessed across all three outcomes and included in the meta‐analysis.

All the animal studies used between‐group experimental designs, where random assignment of animals to groups was unclear. The human observational study assessed the effect of prenatal e‐cigarette exposure, as well as potential postnatal exposure through breastfeeding. Due to the quasi‐experimental design, it is not possible to establish a cause‐and‐effect interpretation from the included human study. Risk of bias for the human study was rated as low using the ROBINS‐I tool [25]. No eligible human studies were identified that assessed exposure to nicotine using NRT (e.g., nicotine patches) during pregnancy.

### 3.3 Categorisation of Cognitive Outcomes

**FIGURE 2:**
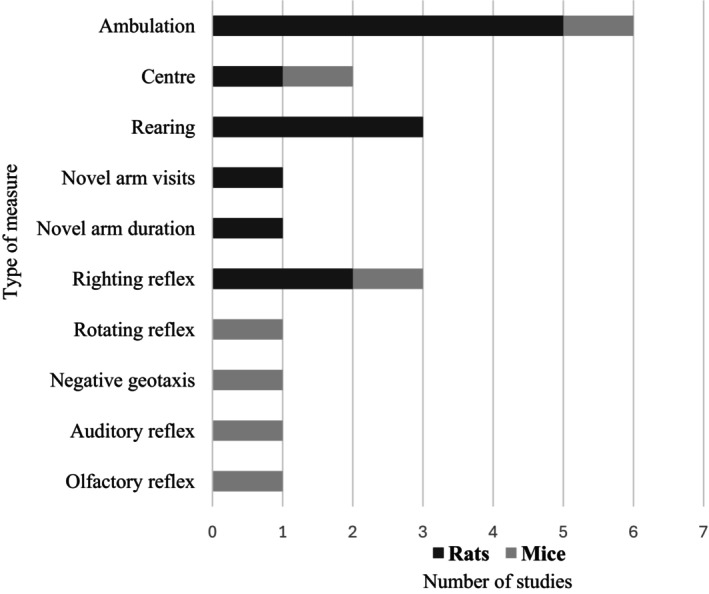
Number of studies using rats and mice across measures. Ambulation, Centre, and Rearing are measures included in the Exploratory behaviour outcome. Novel arm visits and Novel arm duration are measures included in the Spatial memory outcome. Righting reflex, Rotating reflex, Negative geotaxis, Auditory reflex, and Olfactory reflex measures are included in the Reflex development outcome.

In order to review cognitive development, three main outcomes were identified from data extraction of the eligible articles: exploratory behaviour, spatial memory and reflex development. The human study was included in the reflex development outcome only. The number of studies using rat or mouse samples is shown in (Figure 2).

Exploratory behaviour in animals is a complex cognitive process, involving sensory processing, investigation of novel stimuli and information‐gathering [44]. Three different types of exploratory behaviour were measured in the studies included in the review: ambulation (k = 10), centre exploration (k = 2), and rearing behaviour (k = 4). Ambulation is linked to locomotor activity, referring to movement from one location to another measured by the total distance travelled, enabling exploration of an environment [44]. Centre exploration is observed when an animal moves away from the periphery of an open‐field arena and enters a marked centre area to gather information [45]. Rearing is characterised by a vertical movement where a four‐legged animal stands on its hind legs, which can facilitate learning about the spatial environment [46].

Spatial memory refers to the memory system which encodes, stores, recognises and recalls spatial information about the environment [47]. Spatial memory performance, such as the ability to recall a previously visited location, requires hippocampal‐dependent memory [48]. Two types of spatial memory measures were identified for the review and meta‐analysis: novel arm visits in a maze (k = 1) and novel arm visit duration (k = 1).

Reflex development is among the earliest assessments which can be carried out in human newborns and rodent offspring, providing an opportunity to capture disruptions in brain development [49]. In this meta‐analysis, abnormal reflexes are described as an absence or delay of the behaviour. Primitive reflexes are brainstem‐mediated automatic movement patterns which gradually reduce and become voluntary as the CNS matures [50]. Therefore, alterations in the timing of reflex appearance can indicate disruptions in CNS maturation [49, 50], signaling atypical development of motor and sensory systems in the brain, which may negatively affect cognitive and behavioral outcomes [51] in both rodents and humans. As such, pre‐weaning reflex behaviours can serve as early neurodevelopmental markers reflecting processes that support later cognitive development. Longitudinal research of prenatal drug exposure indicates that early neurodevelopmental delays, including alterations in reflex development, are associated with an increased likelihood of adverse cognitive outcomes in childhood [24]. The studies in this review used several measures of reflex development including the righting reflex (k = 5), rotating reflex (k = 1), negative geotaxis test (k = 1), olfactory reflex (k = 1), auditory startle reflex (k = 1) and NBAS assessment of reflexes in human infants (k = 1). The NBAS assessment [52] examines a range of newborn reflexes, including Plantar, Babinski, ankle clonus, rooting, glabella, passive leg tone, passive arm tone, palmer grasp, placing, standing, stepping, crawling, incurvation, tonic deviation, nystagmus, tonic labyrinthine and the Moro reflex. The righting reflex is defined by a pup's ability to roll over onto four paws, with each paw perpendicular to the body, after being placed in the supine position [49]. The rotating reflex and negative geotaxis assess rotation from a downward to upward position on an inclined surface [33]. The olfactory reflex test measures scent discrimination and ability to orient towards an odorous smell, whereas the auditory startle reflex measures behavioural reactions, for example, a sudden body arch, to an unexpected sound [41].

### 3.4 Exploratory Behaviour

**FIGURE 3:**
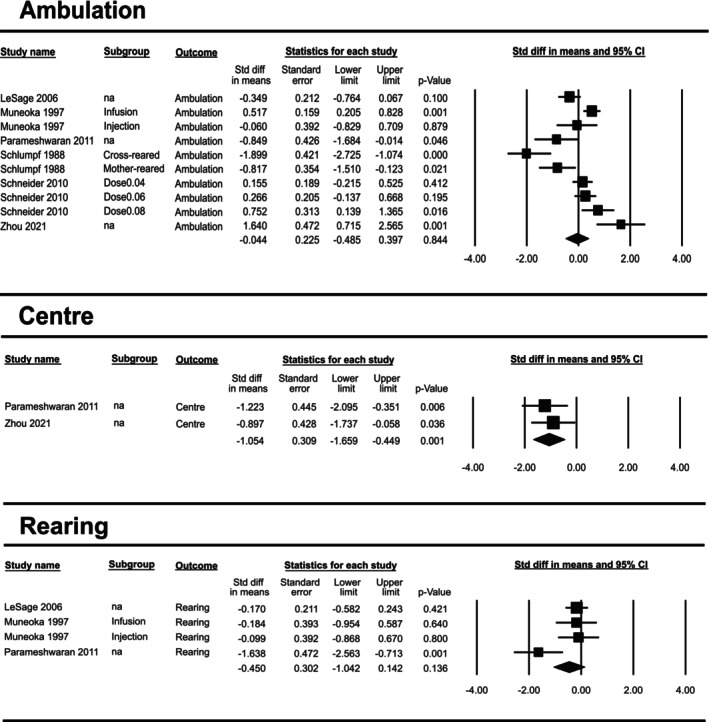
Forest plot of meta‐analysis for the exploratory behaviour outcome. Squares represent individual study effect sizes. Diamonds represent the overall effect sizes. na: Non‐applicable, no sub‐group within study.

The outcome of exploratory behaviour refers to three measures included in the animal studies eligible for the review and meta‐analysis (ambulation, centre exploration and rearing). Forest plots are shown in (Figure 3).

#### 3.4.1 Ambulation

Across all studies, ambulation was the most common measure (k = 10). There was evidence of heterogeneity in the effect sizes (Q = 61.444, *p* < 0.001), *I* ^*2*^ = 85.352%); therefore, the random effects model is reported, which was not significant (*d* = −0.044; 95% CI [**−**0.485, 0.397], Z = −0.197, *p* = 0.844, k = 10). A sensitivity analysis revealed that the results did not vary significantly based on separate exclusion of each study. Prenatal exposure to nicotine, and in the case of one study [41] prenatal and postnatal exposure via feeding, is not significantly related to higher or lower rates of ambulation in offspring. Of the 10 effect sizes in the measure, three reported effects were significantly positive, indicating increased rates of ambulation in the prenatally nicotine‐exposed sample [36, 39, 41], three were significantly negative, [37, 38] —mother‐reared, cross‐reared, and the remaining four showed no significant effect [35, 36]—injection, [39]—dose 0.04, 0.06]. In some studies, the effects seemed to be related to nicotine dosage or method of administration. The study by Schneider et al. [39] found a significant positive effect at the highest exposure dose, and the study by Muneoka et al. [36] only showed a significant positive effect in the infusion rather than injection administration group (See Table 2).

#### 3.4.2 Centre Exploration

Three studies measured centre exploration in rodents. However, in one study [35] there were insufficient data available to calculate the effect size for the centre measure. The fixed effects model is reported, as there was no heterogeneity between the effect sizes (Q = 0.278, *p* = 0.598, *I* ^*2*^ = 0%). The combined effect size of centre exploration was significant (*d* = −1.054; 95% CI [−1.659, −0.449], Z = −3.4164, *p* = 0.0006, k = 2). A significant negative effect size indicates prenatally nicotine‐exposed offspring spent less time exploring the centre of a field compared to the non‐exposed offspring (see Figure 3).

#### 3.4.3 Rearing Behaviour

The frequency of rearing was assessed in three studies, with the study by Muneoka et al. [36] reporting effect sizes for two separate sub‐groups differentiated by method of prenatal nicotine administration (infusion or injection) (see Figure 2). Evidence of heterogeneity was found in the measure (Q = 8.752, *p* = 0.033, *I* ^2^ = 65.722%), therefore the random effects model is reported. The combined effect size was non‐significant (*d* = −0.4503; 95% CI [−1.0422, 0.1416], Z = −1.4911, *p* = 0.1359, k = 4), and a sensitivity analysis indicated no significant variation if a particular study was removed. Only one study reported a significant effect in this measure [37], however, we were unable to identify consistent differences between the studies that could account for this finding. The overall result indicates that there is no significant difference in rearing behaviour between offspring prenatally exposed to nicotine and those not prenatally exposed.

### 3.5 Spatial Memory

In this meta‐analysis, the outcome of spatial memory was determined through rodent performance in the Y‐maze. Only one study [37] examined spatial memory in prenatally nicotine‐exposed pre‐weanling rats, where performance was characterised by two measures, the number of visits to a novel arm in the Y‐maze and novel arm visit duration. The effect size from this single study was found to be negative and significant (*d* = −1.117; 95% CI [−2.112, −0.123], Z = −2.202, *p* = 0.028, k = 1). The prenatally nicotine‐exposed rodents showed lower frequency of visits and duration in the novel arm. Results indicate that spatial memory is impaired in prenatally nicotine‐exposed animal offspring, where inability to discriminate a novel from previously visited maze arm was demonstrated.

### 3.6 Reflex Development

**FIGURE 4:**
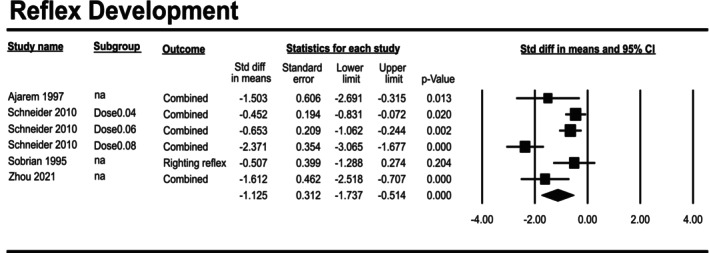
Forest plot of meta‐analysis for the reflex development outcome. Squares represent individual study effect sizes. Diamonds represent the overall effect sizes. na: Non‐applicable, no sub‐group within study.

One human study [34] and four animal studies [33, 39, 40, 41], one of which included three different sub‐groups of varying nicotine exposure dosages [39], measured reflex development in prenatally nicotine‐exposed offspring (Figure 4). To maintain methodological homogeneity, the human observation study [34] which was a quasi‐experimental design was analysed separately and not included in the pooled effect size for this outcome. Reflex assessment in the animal studies varied from righting reflex [33, 39, 40], rotating reflex [33], negative geotaxis [39], auditory and olfactory reflex [41], with righting reflex being the most common measure across the studies. There was evidence of heterogeneity in the effect sizes (Q = 28.186, *p* < 0.001, *I* ^*2*^ = 82.260%), therefore the random effects model is reported. The pooled effect size for reflex development in the animal studies showed a large, statistically significant effect (*d* = −1.126; 95% CI [−1.737, −0.514], Z = −3.608, *p* < 0.001, k = 6; fail‐safe *N* = 92). A sensitivity analysis revealed that results were robust, whereby any of the studies included were not disproportionately impacting the overall effect size. All studies except one [40], indicated a significant negative effect in this outcome see (Figure 3). This could be attributed to age of pups at time of testing or the study aims and design, with this study including multiple treatment exposure groups, compared to the other studies included in the meta‐analysis which solely focused on the effect of nicotine. The human study assessed reflex development via the NBAS reflex measures [34] and was analysed independently in this outcome. This single study demonstrated a significant negative effect (*d* = −1.857; 95% CI [−2.628, −1.087], Z = −4.723, *p* < 0.001, k = 1). Infants exposed to e‐cigarette nicotine exhibited significantly poorer reflex performance compared to non‐exposed infants. Overall, findings from both human and animal studies demonstrate that prenatal nicotine exposure is associated with the delay or absence of postnatal reflex development.

## 4 Discussion

The aim of this systematic review and meta‐analysis was to examine if prenatal exposure to nicotine influences early cognitive development. Overall, results of the meta‐analysis demonstrate that nicotine intake during pregnancy is associated with differences in some domains of postnatal development in humans and animals.

The review focused on three outcomes: exploratory behaviour, spatial memory and reflex development. Nine studies conducted on human and animal samples, which compared offspring that had been exposed to nicotine prenatally with a non‐exposed control group, were included. Results indicated associations between prenatal nicotine exposure and impaired spatial memory, reflex development, and centre exploration, a measure included in the exploratory behaviour outcome. The largest negative effect was observed in reflex development (*d* = −1.126, *p* < 0.001). However, no significant differences were observed in the rate of ambulation and rearing behaviour in offspring.

For the exploratory behaviour outcome, the systematic review assessed three measures of ambulation, centre exploration and rearing represented across six studies. We found no significant pooled effects of prenatal nicotine exposure and rates of ambulation or rearing in rodents. However, nicotine exposure during pregnancy did significantly reduce the duration of centre exploration, compared to a non‐exposed group. The studies which measured rates of ambulation included in the review did not highlight a consistent pattern of ambulatory activity in nicotine‐exposed offspring, with some studies reporting increased rates of ambulation and other studies showing a reduction compared to a non‐exposed group. The measure was highly heterogenous, suggesting variance between studies may account for differences observed in rates of ambulation. However, there were an insufficient number of studies which could be included in the meta‐analysis to further explore the results through moderation analysis. One possible explanation for the differences observed in ambulation of nicotine‐exposed rodents may relate to different methods of nicotine administration or age of pups at testing. For example, offspring demonstrated higher rates of ambulation if nicotine was administered orally through drinking water during pregnancy [39, 41]. Furthermore, in the context of one included study [36], two administration routes of injection or infusion were tested in independent samples. This study showed that prenatally nicotine‐exposed pups in the injection group demonstrated a significant reduction of ambulation on postnatal day 14, compared to prenatally nicotine‐exposed pups in the infusion group, who showed higher rates of ambulation compared to the injection administration group and non‐exposed group. One potential reason for the observed difference of offspring ambulation in the two sub‐groups of nicotine administration may be due to maternal factors. Frequent drug injection can cause maternal stress in rodents, triggering the release of stress hormones [53], known to highly influence post‐birth offspring behaviour [54]. This may also provide an explanation for the higher ambulation rates observed in the studies with oral routes of nicotine administration [39, 41]. Therefore, maternal factors cannot be ignored when evaluating postnatal outcomes, as variables such as maternal stress can impact behaviour of offspring, with this effect also observed in human studies [55].

However, the remaining studies in the ambulation measure which administered nicotine via infusion all demonstrated decreased levels of ambulation in nicotine‐exposed offspring, apart from the infusion administration sub‐group in the study by Muneoka et al. [36]. It could be argued that the conflicting non‐significant results may be attributed to the heterogeneity of experimental approaches among the studies, such as how ambulation is categorised and measured, rodent environment acclimatisation, and the duration of recorded field activity in the study methodology. For example, in the study by Muneoka et al. [36], rodent ambulation rates were recorded for 3 min, compared to 10 min in the study by Parameshwaran et al. [37]. Thus, ambulation may not be an accurate measure of the effect of nicotine exposure on activity levels and exploratory behaviour overall.

In a novel environment, vertical activity is described as exploratory rearing whereby rodents gather information [46]. Although centre exploration is often linked to anxiety‐like traits in animal studies [37], it also assesses behavioural stimulus‐seeking exploration [56]. These measures contrast with ambulation, which generally also includes behaviours of spontaneous activity or exercise that are unrelated to exploration [57]. Two studies in the meta‐analysis showed a significant reduction in centre exploration in nicotine‐exposed rodents, with a third study reporting the same pattern, but without sufficient data for inclusion in the meta‐analysis [35]. Three studies in the meta‐analysis reported reduced frequency of rearing in nicotine‐exposed offspring compared to a non‐exposed group; however, this was not statistically significant. Although rearing and centre exploration may be more reliable indicators of exploratory behaviour compared to ambulation, the effect of prenatal nicotine exposure on rearing was non‐significant, and only two studies were included in the combined effect size for centre exploration; hence, this result should be interpreted with caution. Additional research employing consistent methodologies is needed to further assess the impact of prenatal nicotine on exploratory behaviour in animals.

Spatial memory was reported in one study [37] and showed that prenatal nicotine exposure was associated with spatial memory deficits in offspring. Prenatally nicotine‐exposed rats displayed impaired recognition for novelty, compared to non‐exposed rats, demonstrating a large effect size (*d* = −1.117, *p* < 0.028). The hippocampus is a critical brain region involved in memory and is modulated by nAChRs [58]. Premature activation of nAChRs in the developing brain can occur due to the ability of nicotine to stimulate nAChRs and disrupt neurodevelopmental events that are usually actioned by acetylcholine, altering the trajectory of cognitive development and behaviour [11, 59]. The results from one study included in this review support the possibility that postnatal spatial memory deficits may be caused by the damaging effects of early nicotine exposure on the hippocampus. However, as this finding is based on a single study, further research is required to support the conclusion.

Reflex development, measured in five studies, showed the largest negative effect of prenatal nicotine exposure. Premature activation of nAChRs, which are involved in critical processes of CNS development [10], can result in abnormal cortical wiring and functioning, causing neurodevelopmental delays or impairment of reflex behaviours [49]. Reflex impairments were observed in the animal studies and in one human study, compared to offspring not prenatally exposed to nicotine. Despite the heterogeneity in the outcome, possibly reflecting variable reflex tests included in the animal studies, the overall effect size was highly significant. All studies demonstrated a negative effect; however, one reported a statistically non‐significant result [40]. One possibility for this may be the age of pups at which the righting reflex test was conducted, whereby in the study by Sobrian et al. [40] this reflex was measured on postnatal day three, whereas in the study by Ajarem and Ahmad [33], the righting reflex was assessed on the first postnatal day. Additionally, the non‐significant result in this study may reflect differences in study design. In particular, Sobrian et al. [40] included exposure groups of other drug substances such as cocaine, and therefore the effect of nicotine exposure was not the primary focus of the study.

While further analyses were not possible due to the small number of studies which were eligible for inclusion in the review and meta‐analysis, abnormal reflexes were also observed in groups following indirect continuous after‐birth nicotine exposure through maternal feeding, alongside prenatal exposure, in one human and one animal study [34, 41]. This emphasises that nicotine intake during pregnancy, as well as after birth through nursing, may affect postnatal development and hence, the potential impact of exposure across both periods should be acknowledged. Notably, the human study included in this review also reported abnormal reflexes in infants prenatally exposed to cigarettes [34], suggesting that the adverse effects of smoking during pregnancy on reflex development can be partly attributed to nicotine. Although human research directly investigating the cognitive effects of prenatal nicotine exposure is limited, causal inference is restricted by reliance on naturally occurring groups of self‐reported maternal nicotine intake during pregnancy. Nevertheless, emerging studies show findings consistent with those observed in animal research. Therefore, if cognitive outcomes are not considered in the standard of comparing nicotine delivery system use to cigarette smoking and non‐smoking in pregnancy, potential negative effects of prenatal nicotine exposure may not be detected until later development. This highlights the importance of conducting further research specifically evaluating the cognitive impacts of prenatal nicotine exposure and warrants caution for use of any nicotine delivery systems during pregnancy.

### 4.1 Strengths and Limitations

This systematic review and meta‐analysis advance our understanding of the impact of nicotine exposure during pregnancy on postnatal cognitive outcomes. To identify all relevant academic articles in this area, database and manual reference list searches were carried out, and sensitivity analyses were conducted to detect studies which could be disproportionately affecting the results. Our findings indicate that across all three outcomes, prenatal nicotine exposure showed the strongest negative association with postnatal reflex development. Exposure to nicotine during pregnancy also had a significant negative impact on postnatal centre exploration and spatial memory, compared to non‐exposed rodents. However, these effects were observed in a small number of studies and should be interpreted with caution. One limitation of the current review is the lack of human research focusing on nicotine exposure via nicotine delivery systems, where the inclusion of animal research investigating the effects of prenatal exposure can suffer from assessing equivalent behaviours in animals and humans. However, in the reflex development outcome, a consistent negative effect was observed in both human and animal studies using quasi‐experimental and experimental designs. Another limitation of the meta‐analysis was the inability to conduct further analyses. Although meta‐regression and sub‐group analyses were planned prior to conducting the review, this was not possible due to the small number of studies eligible for inclusion. As a result, this restricted the report of potential variations across different studies, such as nicotine exposure duration or dosage, which may have influenced postnatal outcomes. Thirdly, extracting the effect sizes for the earliest available postnatal age from the included studies may have limited the range of the review. However, identifying a common age across all studies was not possible due to age variation. By focusing on the pre‐weaning period, this systematic review captured the earliest developmental impact of nicotine exposure.

## 5 Conclusions

This systematic review and meta‐analysis provide evidence of an association between prenatal nicotine exposure and early cognitive and neurodevelopmental impairments, particularly in postnatal reflexes and spatial memory outcomes. Given the guidance on nicotine delivery systems and increasing prevalence of e‐cigarette use in pregnancy, advocated as a safer alternative to cigarettes, informing on the potential consequences of early nicotine exposure on foetal and postnatal health is essential. The review highlights that future research needs to include assessment of cognitive outcomes, and move beyond the focus on physical birth outcomes and comparisons to traditional cigarette smoking when considering the safety of nicotine delivery system use during pregnancy.

## Author Contributions

**Nadja Reissland:** conceptualization, methodology, supervision, writing – review and editing, validation. **Deimantė Baguckaitė:** conceptualization, methodology, writing – original draft, writing – review and editing, formal analysis, validation, investigation, visualization. **Judith Covey:** conceptualization, methodology, supervision, writing – review and editing, formal analysis, validation, resources.

## Funding

This work was undertaken as part of a doctoral thesis and was supported by a fellowship from Durham University.

## Conflicts of Interest

The authors declare no conflicts of interest.

## Data Availability

The data that support the findings of this study are available from the corresponding author upon reasonable request.
